# Recent Advances in Functionalized Gold Nanoprobes for Photoacoustic Imaging Analysis of Diseases

**DOI:** 10.3390/s26010203

**Published:** 2025-12-28

**Authors:** Zhiwan Huang, Hanying Ye, Haiting Cao, Yao Ma, Kecheng Lou, Yao He, Binbin Chu

**Affiliations:** 1 Suzhou Key Laboratory of Nanotechnology and Biomedicine, Institute of Functional Nano & Soft Materials (FUNSOM), Collaborative Innovation Center of Suzhou Nano Science and Technology (NANO-CIC), Soochow University, Suzhou 215123, China; 20254214070@stu.suda.edu.cn (Z.H.); 20254214069@stu.suda.edu.cn (H.Y.); haitingcao@suda.edu.cn (H.C.); 20244214072@stu.suda.edu.cn (Y.M.); 20244214074@stu.suda.edu.cn (K.L.); 2 Macao Translational Medicine Center, Macau University of Science and Technology, Taipa 999078, Macau SAR, China; 3 Macao Institute of Materials Science and Engineering, Macau University of Science and Technology, Taipa 999078, Macau SAR, China

**Keywords:** gold nanoprobes, functionalization, nanocomposite, nanoaggregate, photoacoustic imaging, diseases imaging

## Abstract

Photoacoustic imaging (PAI) integrates the high-contrast merits of optical imaging with the high-spatial-resolution advantages of acoustic imaging, enabling the acquisition of three-dimensional images with deep tissue penetration (up to several centimeters) for in vivo disease detection and diagnosis. Among various photoacoustic nanoagents, gold nanomaterials (GNMs) have been widely explored for the PAI-based imaging analysis and photothermal therapy of diseases, owing to their strong near-infrared (NIR) absorption, which can generate distinct photoacoustic signals in deep tissues. This review focuses on recent advances and achievements in the development of functionalized gold nanoprobes, including Janus gold nanoprobes, gold nanocomposite probes (such as functionally coated GNMs and GNMs-loaded nanocarriers), and gold nanoaggregate probes (e.g., pre-assembly of GNMs and in situ aggregation of GNMs). The multifunctionalization of GNMs can enhance their PAI performance by shifting absorption to the NIR-I and NIR-II regions, while simultaneously imparting additional functionalities such as targeted delivery to disease sites and specific responsiveness to disease biomarkers. These features can render functionalized GNMs-based nanoprobes highly suitable for PAI-based analysis and the precise detection of various pathological conditions, including bacterial infections, tumors, kidney injury, and disorders affecting the ocular, gastrointestinal, cardiovascular, visceral, and lymphatic systems. Finally, this review provides a concise summary of biosafety evaluation and outlines the current challenges and future perspectives in optimizing the GNMs-based PAI methods, highlighting their potential to enhance the rapid and precise diagnosis of diseases in the future.

## 1. Introduction

With the rapid development of, and continuous advancements in, modern biomedical and medical technology, the concept of precision medicine has become increasingly established [1,2,3]. Precision medicine seeks to develop targeted and optimized therapeutic strategies based on the unique characteristics of each patient, enabling personalization of the entire medical process and thus maximizing treatment efficacy [1,2,3]. Of particular significance is precision biomedical analysis, especially precision biomedical imaging, which supports accurate disease diagnosis by providing clear and detailed visual information [4,5,6]. Real-time and dynamic imaging facilitates image-guided interventional therapies, allowing for precise interventions, while the long-term monitoring of disease progression enables early detection and prevention [7]. Currently, these widely used clinical imaging modalities mainly include fluorescence imaging [7,8], ultrasonic imaging (i.e., grayscale ultrasound (GSUS), Doppler ultrasound (DUS), and contrast-enhanced ultrasound (CEUS) imaging method) [9,10,11,12], X-ray imaging [13], magnetic resonance imaging (MRI) [14], computed tomography (CT), positron emission tomography-CT (PET-CT), and single-photon emission computed tomography (SPECT) [15]. Among these, the photoacoustic imaging (PAI) method represents a hybrid modality that can combine optical and ultrasonic imaging principles, integrating the high contrast of optical signals with the deep penetration and high spatial resolution of ultrasonic signals [7,16,17,18]. In PAI technology, short-pulsed laser irradiation can induce transient thermoelastic expansion in the optically absorbing tissue, generating the acoustic waves that are detected by ultrasound transducers to amplify the signals [19]. Because the generated ultrasound signals bypass the strong scattering effects associated with photons in biological tissues, PAI is suitable for the effective visualization of deep tissue structures [19,20,21]. Therefore, PAI strategy has gained increasing recognition among clinicians and researchers due to its advantages, including high spatial resolution, substantial penetration depth, excellent imaging contrast, and a favorable biosafety profile. Notably, the PAI method overcomes the inherent depth limitation of conventional optical imaging (~1 mm), achieving imaging depths of up to ~50 mm with micrometer-level resolution. Furthermore, PAI surpasses traditional ultrasound imaging in sensitivity by harnessing intrinsic optical absorption contrasts, thus enabling label-free detection of critical physiological parameters, including blood oxygen saturation [22]. Owing to the above-mentioned advantages, the PAI method has been successfully applied in the diagnosis and monitoring of bacterial infections (e.g., differentiating bacterial from non-bacterial etiologies), tumors (e.g., identifying neovascularization and tumor margins), and diseases of the ocular, gastrointestinal, cardiovascular, and lymphatic systems (e.g., tracking hemodynamic changes) [7,23,24,25,26,27].

Gold nanomaterials (GNMs) are among the most widely utilized exogenous photoacoustic contrast agents in the PAI method because of their tunable optical properties [7,28]. Their performance is primarily governed by localized surface plasmon resonance (LSPR), a phenomenon in which free electrons within GNMs undergo collective oscillation upon excitation by light at a specific resonant frequency, resulting in enhanced optical absorption [7,27,29]. By modulating the size and shape of GNMs (e.g., gold nanoparticles (AuNPs) [30], gold nanorods (AuNRs) [31], gold nanoflowers (AuNFs) [32], gold nanostars (AuNSs) [33], gold nanotriangles (AuNTs) [34], and gold nanosheets (AuNShs) [35]; etc.), their LSPR peak can be precisely tuned to target wavelengths, particularly within the near-infrared (NIR) region, thereby optimizing their utility for deep-tissue imaging. In addition to their role as contrast agents, GNMs exhibit significant photothermal conversion capability and colloidal stability, making them promising candidates for use as photosensitizers in photothermal therapy (PTT) [29]. Despite these advantages, several critical challenges hinder their in-depth biomedical applications. A key limitation is the structural instability of these agents under repetitive laser irradiation, which compromises their efficacy in longitudinal PAI analysis. Moreover, many GNMs exhibit relatively weak optical absorption within the NIR biological window, leading to suboptimal photothermal conversion efficiency [27,28,29,30]. The conventional anisotropic structures of GNMs are prone to morphological degradation, typically transforming into spherical configurations under the pulsed laser exposure at standard PAI intensity, resulting in the swift attenuation of NIR absorption and further limiting their applications in sustained monitoring. As the particle size increases, the scattering cross-section becomes dominant over absorption, reducing the amount of light energy converted into heat and consequently diminishing both photothermal efficacy and photoacoustic signal generation [27,28,29,30]. A fundamental trade-off exists between high-absorption cross-sections for strong photoacoustic signals and high photothermal conversion efficiency for effective PTT, making simultaneous optimization difficult [27,28,29,30]. Efforts to improve stability often reduce optical sensitivity, also creating a key design challenge. Addressing these issues requires advanced nanomaterial engineering to balance optical performance, thermal efficiency, and long-term stability.

**Figure 1:**
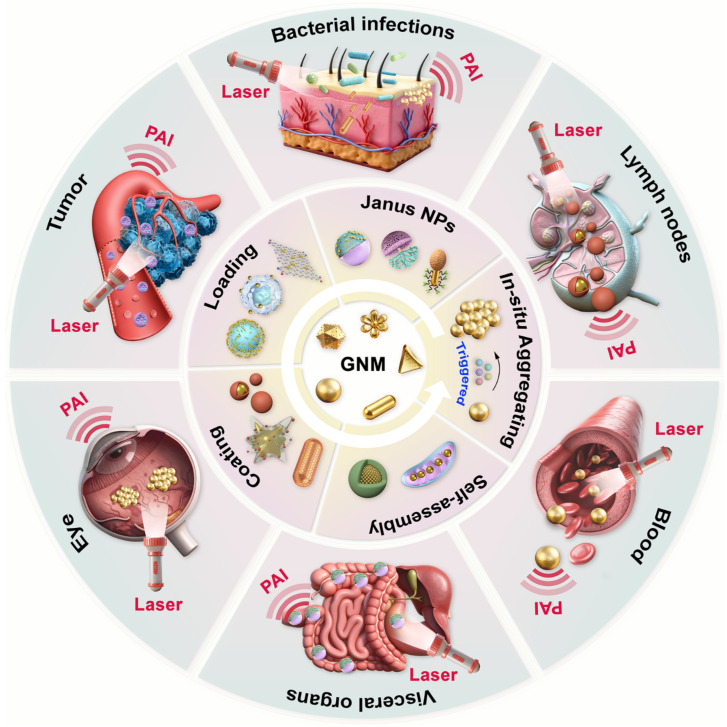
Schematic illustration of functionalized gold nanoprobes for photoacoustic imaging analysis in the diagnosis of various diseases, including bacterial infections, tumors, and ocular, gastrointestinal, blood, and lymphatic disorders. To meet the demands of disease imaging and diagnostic applications, functionalized gold nanoprobes are engineered through the rational design and construction of Janus gold nanoprobes, gold nanocomposite probes (i.e., functionally coated GNMs, and GNMs-loaded nanocarriers) and gold nanoaggregate probes (i.e., pre-assembled aggregation of GNMs and in situ aggregation of GNMs). The term GNMs encompasses various types of morphologies and structures such as AuNPs, AuNRs, AuNFs, AuNSs, AuNTs, and AuNShs.

In this review, we provide a comprehensive review of recent advances in the developments of functionalized gold nanoprobes and their biomedical applications in PAI for the analysis and diagnosis of various diseases over the past decades (Figure 1). First, we focus on the design and fabrication of Janus gold nanoprobes, anisotropic nanostructures comprising at least two chemically distinct domains, which enable PAI for the in vivo analysis of tumors and lymph nodes (Figure 1). Second, we discuss gold nanocomposite probes without anisotropic nanostructures, encompassing functionally coated GNMs and GNMs-loaded nanocarriers, which are applicable to the PAI-based detection of diseases, particularly renal injuries and tumors (Figure 1). Third, we introduce the specific engineering of gold nanoaggregate probes formed either through pre-assembly of GNMs or the in situ stimulus-triggered aggregation of GNMs, highlighting their applicability in PAI diagnosis of tumors, lymph node metastasis, ocular diseases (e.g., retinal pigment epithelium damage and choroidal neovascularization (CNV), etc.), and various bacterial infections (Figure 1). Finally, we summarize biosafety evaluations of these gold nanoprobes, describe the advantages of multifunctional GNM-based platforms in PAI analysis, and outline the current challenges and limitations in future disease detection, thus emphasizing their potential to enhance the speed and accuracy of disease diagnosis.

## 2. Janus Gold Nanoprobes

The concept of Janus particles was first introduced by Pierre-Gilles de Gennes in his 1991 Nobel Prize lecture on soft matter, where he described them as anisotropic nanomaterials [36]. Owing to significant advances in nanotechnology over past decades, Janus nanoprobes have garnered increasing attention in the fields of biological and biomedical imaging and therapy because of their unique structure, comprising at least two chemically distinct domains [37,38,39]. These domains exhibit differential surface chemistry, enabling multifunctionality and novel physical properties derived from structural asymmetry. In comparison with conventional symmetric nanoprobes, the principal advantage of Janus nanoprobes stems from their asymmetric architecture, which facilitates “zonal coordination” and “directional functionality” [37,38,39]. Specifically, first, in traditional probes, functional components, including targeting ligands, therapeutic agents, and imaging moieties, are uniformly distributed across the particle surface, potentially leading to functional interference. In contrast, Janus nanoprobes allow for the precise spatial separation of diverse, and even mutually incompatible, functionalities, such as hydrophobic/hydrophilic, targeting/therapeutic, or imaging/stimuli-responsive elements, onto discrete hemispheres. Second, whereas conventional symmetric nanoparticles tend to undergo isotropic aggregation, often resulting in disordered assemblies, Janus nanoprobes can engage in directional and programmable self-assembly owing to their heterogeneous surface properties. Third, the Janus configuration provides an optimal platform for true “theranostic integration”, enabling the diagnostic functionalities to be localized on one side and therapeutic functions on the other side, hence minimizing cross-interference and maximizing their operational efficiency [40,41,42,43,44].

**Figure 2:**
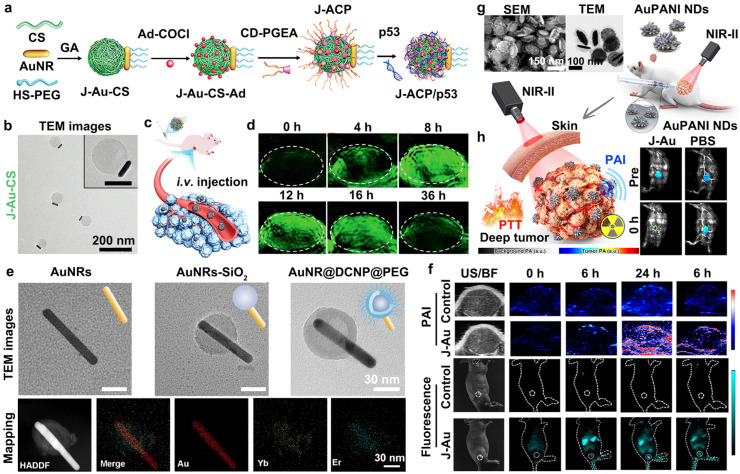
Construction and PAI analysis of Janus gold nanoprobes: (**a**) schematic illustration of the fabrication process for J-ACP/p53 nanoprobes; (**b**) transmission electron microscopy (TEM) and high-resolution TEM images of J-Au-CS nanostructures; (**c**) schematic representation of intravenous administration of J-ACP/p53 for tumor imaging and therapy; (**d**) in vivo PAI analysis of tumors following intravenous injection of J-ACP/p53 probes. The dotted circles indicate the tumor locations. Reprinted with permission from Ref. [45]. Copyright 2021 Wiley-VCH GmbH; (**e**) TEM, high-angle annular dark field-scanning transmission electron microscopy (HAADF-STEM), and elemental mapping images of AuNR@DCNP@PEG; (**f**) in vivo PA and fluorescence imaging of tumors following intravenous injection of AuNR@DCNP@PEG. Reprinted with permission from Ref. [47]. Copyright 2025 American Chemical Society; (**g**) scanning electron microscopy (SEM) and TEM images of AuPNAI NDs; and (**h**) schematic depiction of AuPNAI ND injection for PAI and photothermal therapy (PTT) of tumors. In vivo PAI analysis of tumors after injection of AuPNAI NDs. Reprinted with permission from Ref. [48]. Copyright 2025 American Chemical Society.

Owing to the aforementioned advantages of the asymmetric architecture, a variety of functional materials, including polymers, chitosan, organic semiconducting dyes, iron oxide, silica, and manganese dioxide (MnO_2_), have been employed to enhance the photoacoustic performance of free GNM by stabilizing PA signal integrity, shifting their LSPR from the NIR-I to the NIR-II window, and amplifying PA signal intensity (Table 1) [45,46,47,48,49,50,51]. These asymmetric architectures can also facilitate the integration of multiple imaging modalities and therapeutic functions into a single platform, and improve cellular uptake, and are capable of effectively enhancing the imaging contrast and quality of PAI-based analysis and the detection of various diseases such as tumors (Table 1) [45,46,47,48,49,50,51]. Janus gold nanoprobes can simultaneously exploit the PA signaling and photothermal effects of GNMs, while integrating the drug/gene loading capacity, multimodal imaging functionality, and specific stimulus-responsive behaviors (e.g., to NIR light, temperature, glutathione, pH, and the tumor microenvironment (TME), etc.) conferred by their secondary components. These synergistic attributes render such nanosystems highly promising for precision multimodal imaging diagnosis and effective therapeutic interventions against diseases. In 2021, Xu and co-workers developed a Janus chitosan/gold nanoparticle platform (J-Au-CS) by conjugating polycationic chitosan nanospheres with pre-synthesized PEGylated AuNRs (Figure 2a) [45]. Transmission electron microscopy (TEM) images (Figure 2b) confirmed the successful fabrication of the J-Au-CS nanostructures. The presence of rigid AuNRs on one side of the Janus nanoprobes led to the speculation that the enhanced cellular uptake was attributable to the rigidity imparted by the external AuNRs (Table 1). Subsequent functionalization with both Ad-COCl and CD-PGEA transformed the J-Au-CS system into a NIR-responsive nanocarrier for delivering the tumor suppressor gene p53, with the potential for both PAI analysis and combined PTT/gene therapy. Notably, the Janus nanocarriers exhibited robust responsiveness to NIR irradiation, which triggered the controlled release of p53 and achieved potent gene therapeutic efficacy. As shown in Figure 2c,d, weak PA signals were detected in tumors at 4 h post-intravenous injection; signal intensity increased progressively over time and peaked at 8 h, indicative of efficient tumor accumulation. These findings verify that J-Au-CS can serve as an effective PA contrast agent for visualizing and monitoring tumor progression, as acoustic waves are generated via photothermal effects. In a subsequent study, Song et al. designed a TME-triggered aggregation-enhanced Janus nanoprobe to prolong tumor retention time, suitable for the NIR-II window PAI and boosting photodynamic therapy (PDT) efficacy [46]. Specifically, one hemisphere of AuNRs was coated with MnO_2_, while the opposite side was covalently conjugated to the photosensitizer pyropheophorbide-a (Ppa) via Au–S bonds, namely MnO_2_-AuNR-Ppa Janus nanoprobes. During this process, one side of the AuNRs was protected by a silica shell, whereas the opposite side was coated with MnO_2_. After coating with MnO_2_, the previous silica shell was removed to facilitate the modification of Ppa. Upon accumulation at tumor sites, MnO_2_ was selectively degraded by the endogenous glutathione (GSH), releasing Mn^2+^ that chelated with the multiple Ppa molecules. This interaction induced in situ AuNR aggregation, which significantly amplified the in vivo NIR-II PA signals. The markedly enhanced intratumoral PA signals highlight the potential of these nanoprobes for PAI-guided precision therapy. In 2025, Song et al. introduced a Janus nanostructure-based radiosensitizer to overcome the limitations of conventional tumor radiotherapy [47]. This radiosensitizer was additionally tailored for the NIR-II window (ca. 900–1700 nm) dual-modal fluorescence imaging/PAI analysis, enabling precise and efficient therapeutic guidance [47]. Briefly, the Janus nanoprobes were synthesized via a stepwise approach: (1) selective deposition of silica (SiO_2_) onto one end of high-aspect-ratio AuNRs; (2) in situ growth of a lanthanide-doped down-conversion nanoparticle (DCNP) shell on the SiO_2_ surface; and (3) surface functionalization with biocompatible polyethylene glycol (PEG), yielding AuNR@DCNP@PEG construct (Figure 2e). In this system, the SiO_2_ shell on one side of the AuNRs not only protects the PA performance but also prevents the fluorescence of DCNP from being quenched by AuNRs, preserving dual functionalities in fluorescence imaging and PAI analysis. Meanwhile, the uncoated portion of AuNRs maintains radiosensitization efficacy by generating abundant reactive oxygen species (ROS), which significantly suppress tumor cell growth and metastasis (Table 1). Guided by NIR-II fluorescence and PA imaging (Figure 2f), AuNR@DCNP@PEG was irradiated at the optimal time point of tumor enrichment, which induced substantial ROS generation and further demonstrated outstanding in vivo radiosensitization effects. More recently, Na et al. reported on Janus-type gold nanodiscs (AuPANI NDs) integrated with an asymmetric hierarchical polyaniline architecture (Figure 2g), engineered as multifunctional agents for NIR-II-mediated PTT and multimodal imaging [48]. By the in situ growth of an asymmetric hierarchical polyaniline structure on one side of the gold nanodiscs (AuNDs), the LSPR was effectively shifted from the NIR-I (ca. 800 nm) to the NIR-II (ca. 1047 nm) window (Table 1), enabling the AuPANI NDs to achieve high-performance deep-tissue PAI with a penetration depth of up to ~15 mm (Figure 2h). Furthermore, the inherent modifiability of the AuNDs’ surface allows for efficient radiolabeling via straightforward NOTA conjugation on the opposite side, thus providing an additional strategic advantage in this design and facilitating dual-mode photoacoustic and positron emission tomography (PET) imaging (Figure 2h). Under 1064 nm laser irradiation, these Janus nanoprobes demonstrated excellent photostability and potent therapeutic efficacy in PAI-guided PTT [48]. The one- or two-dimensional architecture of the GNMs provides a highly favorable scaffold for Janus nanoprobes design and fabrication. They have the ability to facilitate the seamless integration of the superior optical and physical properties of GNMs with those of other functional materials, thereby synergistically enhancing the multifunctionality of GNMs and addressing critical challenges in multi-target imaging and therapeutic interventions for complex diseases.

Beyond one-dimensional AuNRs and two-dimensional AuNDs, zero-dimensional AuNPs combined with SiO_2_ or organic–inorganic nanocomposites systems can also serve as versatile building blocks for designing and fabricating Janus gold nanoprobes with distinct functional properties [49,50,51]. Kane et al. demonstrated that gold–silica Janus nanoparticles (GSJNPs) exhibit superior PAI properties compared to gold–silica core-shell nanoparticles, as the asymmetric silica shell not only preserves the photoacoustic and photothermal performance but also maintains the aggregation behavior of bare AuNPs [50]. Under 700 nm excitation, the PA signal intensity of the Janus nanoparticles was significantly higher than that of the core-shell counterparts. Quantitative analysis based on an average of 100 measurement frames revealed an ~8-fold enhancement in PA signal intensity for the Janus nanoparticles relative to the core-shell nanoparticles. In 2021, Song and co-workers developed a versatile PAI platform by integrating stimuli-responsive AuNPs and organic semiconducting dyes into discrete domains of Janus nanocomposites (JNCPs) [51]. Guided by theoretical simulations, they further concluded that the formation of diverse nanostructures within JNCPs stems from entropy-driven equilibrium in the system. Notably, when semiconducting materials (e.g., IR dyes) are adsorbed onto Au nanostructured surfaces, their light absorption efficiency is often compromised, resulting in attenuated PTT efficacy and variable PA signals. By exploiting the phase-separated nature of the Janus nanostructure, this cross-interference can be mitigated by isolating IR dyes and Au nanostructures into separate compartments. In this configuration, the AuNP assembly generates a pH-responsive PA signal, while the semiconducting molecule acts as an internal PA reference, thereby enabling the ratiometric PAI for precise pH sensing in biological environments. Moreover, Liz-Marzán and colleagues also developed a class of gold–iron oxide Janus magnetic-plasmonic nanoparticles (JMNSs) as a nanoprobe [49]. These JMNSs feature two chemically distinct surface domains, which facilitate efficient surface functionalization. The researchers also indicated the utility of JMNSs across multiple imaging modalities, including MRI, CT, PAI analysis, bright- and dark-field optical microscopy, and surface-enhanced Raman scattering (SERS) imaging. Compared to conventional core-shell NPs, the Janus architecture offers notable advantages, most notably enhanced accessibility to the Fe_3_O_4_ surface, which contributes to higher r_2_ relativity values and effective Prussian blue staining. This structural asymmetry renders Janus nanoprobes particularly well-suited for biomedical applications, where spatially segregated functional groups can mediate specific interactions with biological systems, analogous to protein-ligand recognition.

Collectively, asymmetric encapsulation shells can not only enhance the intrinsic PA and photothermal properties of GNMs but also preserve the inherent characteristics of the exposed gold nanostructures, thus improving performance in PAI analysis (Table 1) [45,46,47,48,49,50,51]. Dual-terminal heterogeneous modifications enable the seamless integration of distinct functionalities; rather than interfering with each other, these functional moieties work synergistically to increase probe controllability and facilitate ratiometric PAI analysis for precise diagnosis [45,46,47,48,49,50,51]. Furthermore, site-specific modifications at both terminals can introduce additional imaging and therapeutic functions to a single nanosystem, ultimately achieving theranostic integration. Therefore, the unique features of the Janus nanoparticle architecture, particularly the presence of two functionally distinct surface regions, highlight its significant potential for the multi-target imaging analysis and therapeutic applications across diverse biomedical fields.

## 3. Gold Nanocomposite Probes

To enhance the PA performance and multifunctionality, such as PA signal amplification, tumor targeting, and responsiveness to pathological conditions, of gold nanoprobes, various multifunctional surface coatings have also been employed. As summarized in Table 2, these coatings, including polydopamine (PDA) [52,53,54,55,56,57], hyaluronic acid (HA) [58,59,60,61], silica/SiO_2_ [61,62,63,64,65,66,67,68], and diverse metal-containing shells [69,70,71,72], have been utilized to design and construct functionally coated GNMs-based probes for PAI applications. Furthermore, a range of nanocarriers has been explored to load GNMs for combined PAI analysis. These functional nanocarriers contain DNA origami nanoparticles (DONs) [73]; two-dimensional nanomaterials, including molybdenum disulfide (MoS_2_) [74]; graphene oxide (GO) [75,76]; antimonene nanosheets [77]; MXene nanosheets [78]; silicon-based nanostructures, including silicon nanorods (SiNRs) [79] and silicon nanowires (SiNWs) [80]; magnetic nanoparticles (e.g., Fe_3_O_4_) [81,82]; polymers [83,84,85,86,87]; nanogels [85,88]; microbubbles [89,90], and biological cells such as stem cells and platelets [91,92,93]. This section provides a brief review of these gold nanocomposite probes, including functionally coated GNMs and GNMs-loaded nanocarriers, and their applications in PAI-based disease diagnosis, with a particular focus on kidney injuries and tumors.

### 3.1. Functionally Coated GNMs

Driven by the rapid advances in GNMs, researchers have found that bare GNMs undergo shape deformation, known as melting effects, under laser irradiation, leading to the loss of their LSPR properties [27,28,29]. The loss of their LSPR properties can reduce PA signal intensity and photothermal efficiency. Challenges, such as inherent toxicity and low PA efficiency in the tissue transparency window, further limit their further use in vivo. To overcome these issues, GNMs are often coated with the various types of functional shells, especially polydopamine (PDA), a synthetic melanin analog [52,53,54,55,56,57]. In 2018, Kuehne et al. developed a simple method to produce gold-core/melanin-shell nanoparticles with tunable shapes, including spheres, nanostars, and nanorods [55]. The method uses dopamine auto-oxidative polymerization to form crosslinked, insoluble melanin layers on the AuNPs for PA imaging. Melanin coating can enhance PA contrast more than pure melanin or unmodified AuNPs, and simultaneously improves biocompatibility of the prepared GNMs. Notably, melanin-coated AuNRs show strong enhancement of PA signals. Jokerst et al. also designed and created PDA-coated AuNRs (AuNRs@PDA) for stable, high-performance theranostic applications [53]. AuNRs@PDA generate ~3-fold higher PA signals and 10% greater photothermal efficiency than bare AuNRs, thanks to improved optical absorption and a 2.4-fold increase in thermal conversion. PDA coatings enhance light harvesting, reduce energy loss, and induce a red shift in plasmon resonances. Moreover, Coy et al. indicated that the LSPR peak of the AuNRs/PDA can be tuned from ~750 to ~950 nm by adjusting the thickness of PDA shell from 0–30 nm (Table 2) [54]. These results revealed the promise of PDA-coated GNM, with the potential for improved PAI analysis.

Hyaluronic acid (HA) is highly biocompatible, biodegradable, and biologically active, with specific affinity for the CD44 receptor that is overexpressed on tumor cells [58,59,60,61]. This enables HA-functionalized nanoparticles to target drug delivery, proteins, and genes selectively via CD44-mediated endocytosis. Au-HA nanocomposites have been developed for targeted PAI and cancer therapy. For instance, HA-cloaked AuNRs-PGED/pDNA complexes (AP/pDNA-HA) significantly supported prolonged circulation and tumor-specific targeting for PAI-guided photothermal and gene therapy [58]. The AuNRs core provides both imaging and photothermal functions, while the HA coating enhances stability, biocompatibility, circulation time, and tumor targeting. Moreover, pH-responsive Schiff base linkages promote efficient endosomal/lysosomal escape. Studies show that the HA-coated GNM nanoprobes could allow for low-dose, infrequent (e.g., weekly) administration with both high efficacy and low toxicity. These results confirm the strong biomedical promise of HA-coated GNM-based PAI platforms [58,59,60,61].

A growing number of researchers have focused on improving PA response and biocompatibility by developing Au-based core-shell nanostructures with the surface coatings such as GO and silica [61,62,63,64,65,66,67,68,75]. Silica coatings are particularly effective due to their ability to lower thermal resistance and stabilize nanoparticle structure [61,62,63,64,65,66,67,68]. These Au-silica core-shell structures generate significantly stronger PA signals than bare GNMs. The enhanced signal arises from thermal expansion of both the silica-coated nanorods and the surrounding liquid, driven by improved thermal conductance at the gold–silica and silica-water interfaces [61,62,63,64,65,66,67,68]. Based on these insights, researchers have created bacteria- or virus-mimetic gold–silica nanocomposites by tuning reaction conditions [64]. Cai et al. developed bacteria-like AuNRs@MSN nanocomposites by uniformly coating AuNRs with the mesoporous silica in an oil-water system, resulting in larger pores and higher surface area [64]. When loaded with drugs like doxorubicin (DOX) or paclitaxel (PTX), these nanocomposites could be employed for multimodal imaging and combined tumor therapy [64]. Notably, Qin et al. achieved a 400% PA signal boost under non-cavitation conditions using a thin silica shell and picosecond laser pulses, compared to the uncoated AuNPs [67]. They further identified two competing processes: transient absorption, which limits photon energy uptake, and electron-phonon energy transfer at the gold–silica interface, which partially counteracts this loss [67].

Various metal-containing shells, including Ag_2_S/Se, Ag, Pd, and MnO_2_, have been used to coat GNM, enhancing their performance in PAI performance [69,70,71,72]. Ag^+^ ions can quench GNM-based PA signals, but signal recovery occurs upon Ag^+^ release. Due to their strong antibacterial properties, Ag^+^ ions and AgNPs enable the targeted treatment of infections [71]. By combining Ag^+^ with AuNRs, Au-Ag and Au-Ag_2_S/Se core-shell structures have been designed for dual-mode PA imaging and therapy for bacterial infections [69,70,71]. These nanostructures are stable under ambient conditions but undergo oxidative etching when exposed to ferricyanide, releasing Ag^+^ and restoring PA contrast, allowing for the non-invasive monitoring of Ag^+^ release in bacterial sites. The released Ag^+^ shows broad-spectrum antibacterial activity. Additionally, palladium-tipped AuNRs (PTA NRs) were synthesized by selectively depositing Pd at both ends of AuNRs, while the uniformly coated Pd-AuNRs (PCA NRs) served as controls [72]. After intravenous injection, PTA NRs accumulated significantly in tumor microvasculature, with PA signals peaking at 12 h, indicating prolonged circulation [72]. These findings indicate the enhanced PA performance of PTA NRs, highlighting their potential for tumor diagnosis via the PAI method.

**Figure 3:**
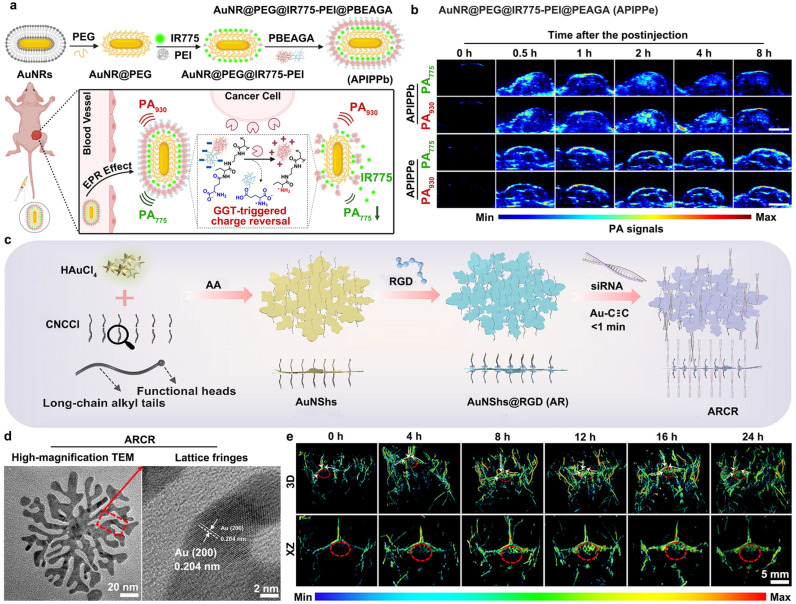
Construction and PAI analysis of multifunctionally coated GNMs: (**a**) schematic illustration of the fabrication process and in vivo PAI evaluation of AuNR@PEG@IR775-PEI@PBEAGA (referred to as APIPPb) probes; (**b**) in vivo PA images of tumor regions following the administration of APIPPb or APIPPe (AuNR@PEG@IR775-PEI@PEAGA, the control probes) at 0, 0.5, 1, 2, 4, 8 h post-injection. Images were acquired at excitation wavelengths of 775 nm and 930 nm. Scale bars, 5 mm. Reprinted with permission from Ref. [96]. Copyright 2025 American Chemical Society; (**c**) schematic representation of the design and construction of AuNSs-RGD-C≡C-siRNA (hereafter referred to as ARCR) probes; (**d**) high-magnification TEM images and corresponding lattice fringes of the synthesized ARCR probes; and (**e**) in vivo PAI analysis of glioblastoma regions in mouse models following administration of ARCR at various time points (0, 4, 8, 12, 16, and 24 h), including 3D and X-Z plane PA images of glioblastoma. Red circles and white arrows indicate the locations of orthotopic glioblastoma tumors and blood vessels, respectively. Reprinted with permission from Ref. [94]. Copyright 2024 American Chemical Society.

Multifunctionally coated GNMs with multilayer structures or distinct functional domains are widely used to enhance PA signals, precisely target lesions, extend blood circulation and tumor retention, as well as to reduce potential toxicity [94,95,96,97,98,99]. Zhou et al. developed APIPPb by coating AuNRs with PEG, IR775-doped PEI, and PBEAGA-a GGT-responsive polymer (Figure 3a) [96]. In tumors, overexpressed GGT cleaves the PBEAGA shell, releasing IR775 and reducing the PA_775_ signals. The PA_930_ signal from the AuNRs’ core remains stable, serving as an internal reference. Over time, PA_775_ decreases at tumor sites while PA_930_ stays constant; in contrast, control probes (APIPPe) show no change at either wavelength. This allows for the quantitative PA imaging and analysis of GGT activity via PA_775_/PA_930_ ratios (Figure 3b). Dai et al. also designed ARCR by modifying AuNShs with c(RGDyC) peptides and siRNA (Figure 3c) [94]. ARCR shows a flower-like structure with clear lattice fringes from the Au (200) plane, confirming proper formation (Figure 3d). After intravenous injection, ARCR accumulates in glioblastoma tumors, with the PA signal increasing over 12 h, enabling the clear visualization of tumors in the brain (Figure 3e). These results show that multifunctionally coated GNMs are suitable for high-contrast, targeted, and quantitative PAI analysis non-invasive tumor detection.

### 3.2. GNMs-Loaded Nanocarriers

**Figure 4:**
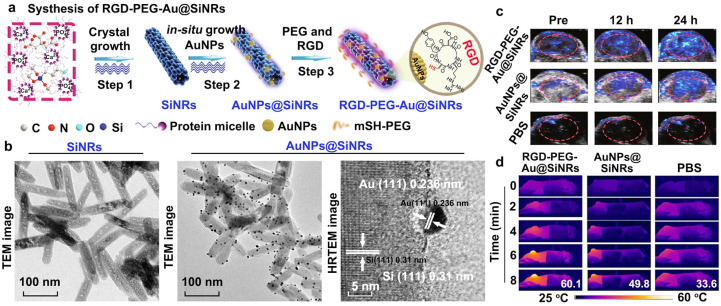
Construction and PAI evaluation of AuNRs-loaded SiNRs (AuNPs@SiNRs): (**a**) schematic illustration of the fabrication process for RGD-PEG-Au@SiNRs nanoprobes; (**b**) TEM and HRTEM images of pristine SiNRs and AuNPs@SiNRs, demonstrating successful loading of AuNPs onto the SiNR surface; (**c**) in vivo PA images of tumor-bearing mice following administration or non-administration of RGD-PEG-Au@SiNRs, showing enhanced contrast at the tumor site; the red circles indicate the tumor locations. and (**d**) in vivo photothermal images of tumor-bearing mice treated with or without RGD-PEG-Au@SiNRs after 8 min of laser irradiation, indicating significant temperature rise in the treated group, attributable to the photothermal conversion capability of RGD-PEG-Au@SiNRs. Reprinted with permission from Ref. [79]. Copyright 2019 Springer Nature Switzerland AG.

Zero-dimensional and one-dimensional nanomaterials, such as Fe_3_O_4_ magnetosomes, Fe_3_O_4_ nanoparticles, SiNRs, and SiNWs, have been used as nanocarriers to load GNMs, forming a core-satellite structure for multifunctional biomedical applications [79,80,81,82]. In situ growth of AuNPs on SiNRs or SiNWs has enabled the fabrication of Si-Au core-satellite nanostructures. He et al. developed AuNPs@SiNRs by depositing AuNPs onto SiNR surfaces, resulting in well-defined core-satellite architectures (Figure 4a) [79]. No AuNPs were observed on the pristine SiNRs; however, AuNPs@SiNRs showed numerous AuNPs with clear lattice fringes corresponding to the Au (111) plane, confirming successful assembly (Figure 4b) [79]. To target CT26 tumors, c(RGDyC) peptides were conjugated to the AuNP satellites, termed RGD-Au@SiNRs. CT26 tumors treated with RGD-Au@SiNRs showed strong PA signals, unlike those treated with non-targeted Au@SiNRs (Figure 4c), demonstrating the effective targeted PAI analysis of tumors. RGD-Au@SiNRs also generated significant photothermal effects, rapidly heating tumors to ~60 °C within 8 min, indicating the potential for PTT (Figure 4d). These results show that Au@SiNRs are suitable for targeted PAI analysis and PTT of tumors [79]. Replacing SiNRs/SiNWs with magnetic nanoparticles, Fe_3_O_4_-Au core-satellite systems have been developed for multimodal imaging and combination therapies [81,82]. These hybrids combine MRI contrast and magnetic enrichment from Fe_3_O_4_ with PA and photothermal effects from loaded AuNPs, enabling combined MRI/PAI imaging and synergistic therapies such as PTT, chemodynamic therapy (CDT), and photodynamic therapy (PDT). Natural Fe_3_O_4_ magnetosomes from the Magnetospirillum magneticum AMB-1 were used to build a magnetic nanoplatform [81]. These magnetosome chassis (MSCs) can be produced at scale via bacterial cultivation. Using a seed-mediated method, AuNPs were grown on MSC membranes to form MSC-Au core-satellite structures. The MSC core provides monodispersity, biocompatibility, MRI contrast, magnetic responsiveness, and CDT capability, while AuNPs add PA and photothermal functions [81]. Importantly, surface gold atoms activate oxygen and promote electron transfer from glucose to oxygen, giving small AuNPs glucose oxidase-like activity. This enables tumor glucose consumption for starvation therapy and generates H_2_O_2_ to enhance CDT. The photothermal effects of AuNPs further supports PA imaging and PTT. Nanogels and microbubbles (MBs) have also been used to carry AuNPs or AuNRs for improved PA contrast and therapy [85,88,89,90]. Liu et al. developed Au@lip MBs-lipid-stabilized MBs encapsulating AuNPs, for ultrasound-responsive PA imaging [89]. Upon ultrasound exposure, these MBs rupture into Au@lip nanoparticle aggregates, which exhibit enhanced near-infrared PA signals due to red-shifted plasmon resonance. By subtracting pre- and post-burst PA images, background signals are removed, making them capable of high-sensitivity, background-free PA imaging, as confirmed in ex vivo and in vivo experiments.

**Figure 5:**
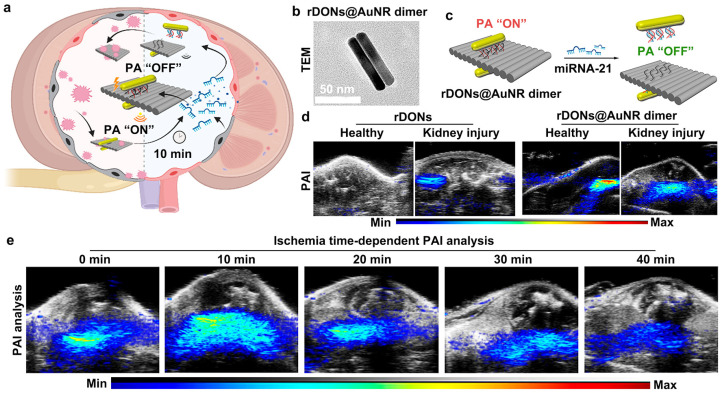
In vivo PAI analysis and detection of acute kidney injury using the AuNRs-loaded rDONs nanoprobes: (**a**) schematic illustration of the rDONs@AuNRs dimer for quantitative PAI detection of acute kidney injury; (**b**) TEM images of the rDONs@AuNRs dimer; (**c**) schematic representation of the detection mechanism of the rDONs@AuNRs dimer for specific imaging of miRNA-21 associated with acute kidney injury; (**d**) in vivo PAI evaluation in healthy mice and mice with acute kidney injury following administration of either the rDONs@AuNRs dimer or bare rDONs; and (**e**) in vivo PAI monitoring during the ischemic process in mice after treatment with rDONs@AuNRs dimer or bare rDONs. Reprinted with permission from Ref. [73]. Copyright 2022 American Chemical Society.

Recent studies have also demonstrated that numerous two-dimensional (2D) nanomaterials, including GO, MoS_2_, MnO_2_, DONs, Ti_3_C_2_ MXene nanosheets, and antimonene nanosheets (AMNSs), have emerged as multifunctional nanoplatforms to load the GNMs for the precision detection and combined therapy of various diseases [73,74,75,76,77,78]. This is attributed to their anisotropic electrical and optical properties, as well as superior chemical characteristics such as facile surface functionalization, high drug-loading capacity, fluorescence quenching ability, and photothermal effects [73,74,75,76,77,78]. Owing to their inherent two-dimensional architecture, most of these two-dimensional nanomaterials have been widely employed as effective nanocarriers for loading AuNPs and/or AuNRs, enabling the construction of novel Au-based nanocomposites that integrate the unique advantages of 2D materials and gold nanostructures. Specifically, AuNR/PrGO composite nanocarriers, comprising partially reduced graphene oxide (PrGO) and AuNRs, have been designed for NIR-induced PDT, PTT, and PAI analysis [76]. When further conjugated with photosensitizers (e.g., Ce6) and tumor-targeting ligands (e.g., folic acid-functionalized PEG polymers), AuNR/PrGO could be employed for the targeted PAI analysis and synergistic PDT/PTT in folate receptor-overexpressing MCF-7 cells. Cheng et al. also developed a novel strategy to precisely control the epitaxial growth of gold nanostructures on MoS_2_ nanosheets (MoS_2_-Au), resulting in both significantly enhanced NIR absorption and good photostability [74]. Following polyethylene glycol (PEG) modification, the resulting MoS_2_-Au-PEG nanoprobes exhibited strong X-ray attenuation and efficient photothermal conversion, demonstrating great potential for enhanced CT and PA imaging in tumors. Moreover, in vivo combination therapy using MoS_2_-Au-PEG achieved synergistic antitumor efficacy through the hyperthermia-induced tumor oxygenation and radiosensitization. Recently, Wang et al. reported a biodegradable, multifunctional nanosystem in which small-molecule photosensitizer IR820 and small-sized AuNPs were loaded onto chitosan-coated AMNSs [77]. This platform simultaneously exhibited outstanding PTT and PDT performance, favorable degradability, robust PAI ability, good biocompatibility, and effective NIR-triggered intracellular synergistic therapy. More recently, He et al. developed a microRNA-21 (miR-21)-responsive nanoantenna composed of AuNRs dimers functionalized with reconfigurable DONs (rDONs), namely rDONs@AuNRs dimer, for early diagnosis and targeted treatment of acute kidney injury (Figure 5a) [73]. Figure 5b clearly illustrates the successful design and assembly of the AuNR dimer structure. miR-21, a known biomarker of acute kidney injury, triggers the release of the AuNR dimer from the rDONs surface, leading to a reduction in PA signal intensity and thus enabling PAI-based detection of acute kidney injury (Figure 5c). As expected, significantly weaker PA signals were observed in mice with acute kidney injury compared to those in healthy mice (Figure 5d). Importantly, a progressively decreasing PA signal was detected during the ischemic progression in mice, allowing for the real-time monitoring of acute kidney injury developments (Figure 5e). These findings confirm the feasibility of using rDONs@AuNRs dimer for PAI-based diagnosis of acute kidney injury [73]. Collectively, these studies provide a rational and versatile approach for constructing gold-based theranostic platforms and expand the potential of gold nanotheranostics in multimodal applications, including multimodal imaging and combinatorial therapies for disease diagnosis and treatment.

Stem cells and platelets have been engineered as biovehicles for delivering GNMs to tumors, leveraging their natural biological properties [91,92,93]. Stem cells are particularly promising due to their intrinsic tumor-tropic ability, while platelets play key roles in inflammation, thrombosis, and cerebral infarction, making them valuable for biomedical applications. These cells have been used to deliver AuNPs, AuNRs, and AuNSs to disease sites. For example, Cheng et al. developed photosensitive mesenchymal stem cells loaded with mesoporous silica-coated gold nanostars (MGNSs) and indocyanine green for imaging-guided photothermal therapy in breast cancer [93]. MGNSs served as stable probes for PA, fluorescence, and photothermal imaging. Their strong photothermal stability enabled real-time, long-term 3D PA imaging of stem cell distribution in tumors, while 3D ultrasound visualized tumor structure. PA results showed the widest stem cell spread in tumors after the 24 h injection, confirmed by 2D fluorescence and photothermal imaging. These findings support the development of cell-based gold nanoprobes for enhanced, targeted tumor imaging.

The multimodal modification of GNMs, through encapsulation, coating, and functionalization, improves dispersibility, stability, biocompatibility, and PA signal intensity, leading to higher image resolution and contrast [52,53,54,55,56,57,58,59,60,61,62,63,64,65,66,67,68,69,70,71,72,94,95,96,97,98,99]. Silica and polydopamine (PDA) shells protect the gold core, ensuring stable PA signals and further leading to a red shift of their LSPR performance (Table 2) [52,53,54,55,56,57,58,59,60,61,62,63,64,65,66,67,68,69,70,71,72]. PEG can extend blood circulation time, increasing GNMs accumulation at target sites and enhancing signal strength and specificity. Biomolecules, such as peptides, proteins, DNA, aptamers, and cell membranes, can be attached to enable specific binding to cancer cells, promoting selective uptake, increasing local concentration, and improving the signal-to-background ratio. Dyes like ICG and Cy5.5 can be adsorbed on GNMs or embedded in coatings, capable of multimodal imaging analysis. The GNMs core acts as a nanoantenna, using plasmon resonance energy transfer to quench fluorescence and boost PA signals, especially in the NIR regions range for deeper tissue penetration. This synergy produces stronger signals than either component alone. Coating type and structure directly influence PA signal stability, specificity, and intensity, guiding the design of precise diagnostic nanoprobes. Loading GNMs onto a nanocarrier also enables the multifunctional theranostic platforms that combine enhanced PA imaging with responsiveness, targeted delivery and combination therapies.

## 4. Gold Nanoaggregate Probes

It has been demonstrated that AuNPs with diameters exceeding 50 nm exhibit strong NIR absorption, which is advantageous for PAI analysis and photothermal effects [100,101,102]. However, small-sized AuNPs are generally preferred for biomedical applications due to their shorter biological half-life and reduced blood residence time [100,101,102]. In order to reconcile this trade-off, strategies involving the smart pre-assembly [103,104,105,106,107,108,109,110,111,112,113,114] or in situ aggregation [115,116,117,118,119,120,121,122,123,124,125,126,127,128,129,130,131,132,133,134,135] of small AuNPs, both in vitro and in vivo, have been explored to induce a red shift in surface plasmon resonance into the NIR region, capable of enhancing both PA signals and photothermal efficacy while retaining the favorable pharmacokinetic properties of smaller AuNPs (Table 3) [103,104,105,106,107,108,109,110,111,112,113,114,115,116,117,118,119,120,121,122,123,124,125,126,127,128,129,130,131,132,133,134,135]. To date, numerous studies have reported the development of various multifunctional gold nanoclusters and nanoaggregates designed for PAI and PTT in cancer, lymph node metastasis, ocular disorders (e.g., retinal pigment epithelium damage and CNV), and bacterial infections [103,104,105,106,107,108,109,110,111,112,113,114,115,116,117,118,119,120,121,122,123,124,125,126,127,128,129,130,131,132,133,134,135].

### 4.1. Pre-Assembly of GNMs

In recent years, scientists have developed a variety of gold nanoaggregates, including chain-like AuNP clusters, spherical AuNPs/AuNRs vesicles, and tunable gold nanostructures (e.g., arcs, rings, ribbons, and vesicles), by inducing the pre/self-assembly of AuNPs or AuNRs through the established stimulus-responsive strategies [103,104,105,106,107,108,109,110,111,112,113,114]. These assemblies have emerged as powerful tools for PAI analysis and effective therapeutic applications in various diseases. Notably, tunable gold nanostructures exhibit enhanced PA signals generation and improved capabilities for imaging-guided synergistic therapy [104]. In 2020, Xu et al. reported a simple and versatile approach combining emulsion-templated and polymer-guided assembly to fabricate a series of structurally controllable gold patterns [104]. The amphiphilic block copolymer (BCP)-functionalized AuNPs (named Au@BCP) were first synthesized via the Au-S bond formation. Subsequently, hexadecyltrimethylammonium bromide (CTAB) was introduced into a chloroform solution containing the as-prepared Au@BCP and free poly (lactic-co-glycolic acid) (PLGA) to form a stable oil-in-water emulsion. Upon the evaporation of the chloroform, gold nanoaggregates with tunable morphologies, such as arcs, rings, ribbons, and vesicles, were obtained by modulating the concentrations of CTAB, PLGA, or Au@BCP. Specifically, increasing the concentration of Au@BCP could lead to progressive structural transitions from arcs to rings, ribbons, and ultimately vesicle-like assemblies [104]. The resulting nanoaggregates demonstrated significant potential for PAI-guided cancer therapy due to their prolonged retention in tumor tissues [104]. Beyond PLGA, nanogels, upconversion and downconversion nanoparticles, and Ag_2_S quantum dots have been used to guide the self-assembly of spherical AuNPs or AuNRs vesicles [109,110,111,112,113,114]. In 2017, Emelianov et al. used PNIPAM nanogels to control AuNR aggregation, forming spherical nanoclusters with stronger PA signals [109]. Later, Gao et al. further developed a pH-responsive vesicle system from thiolated polymer-grafted AuNPs and Ag_2_S QDs [112]. In detail, this system showed promise for multimodal imaging, including PA and NIR-II fluorescence (FL) imaging, in cancer diagnostics [112]. Initially, the assembled AuNP-Ag_2_S vesicles exhibited strong PA signals and quenched NIR-II fluorescence signals, making them suitable for tumor detection via PAI analysis [112]. Upon entering cancer cells, the acidic intracellular environment triggered disassembly of the vesicles, releasing Ag_2_S QDs and restoring fluorescence signals, while PA signals diminished concurrently, enabling in vivo fluorescence imaging of tumors. Importantly, the integration of NIR-II fluorescence and PA imaging facilitated precise monitoring of radiotherapeutic outcomes and minimized unintended damage to healthy tissues, highlighting its potential for advancing precision biomedicine.

In addition to the spherical gold nanoaggregates, the chain-like AuNPs clusters have also been engineered to enhance PA signal intensity and improve imaging performance in disease diagnosis [105,106,107]. In 2021, Zhang et al. reported the development of silica-encapsulated self-assembled gold nanochains (AuNCs@SiO_2_) for applications in precise tumor diagnosis and targeted therapy [105]. The silica coating significantly enhanced physicochemical stability and improved biocompatibility, thus supporting their potential use as a reliable nanoplatform in oncological theranostics. Briefly, the dynamic chain structure of the prepared AuNCs@SiO_2_ enabled absorption red shifting from the visible to the NIR-II region due to plasmonic coupling between adjacent nanoparticles, thereby extending beyond conventional molecular imaging windows and enabling high-sensitivity PA imaging of tumors [105]. In 2021, Paulus and coworkers developed chain-like gold nanoparticle (CGNP) clusters using AuNPs modified with the pentapeptide Cys-Ala-Leu-Asn-Asn (CALNN) and cysteamine ligands [106,107]. The CGNP surface was further conjugated with RGD peptides (yielding CGNP-RGD) and labeled with the near-infrared fluorescent dye indocyanine green (ICG), finally forming the ICG-CGNP-RGD probes. These multifunctional photoacoustic probes were incorporated into ARPE-19 cells to enable multimodal imaging and detection of retinal pigment epithelial damage. Following subretinal injection, the labeled cells generated both PA and FL signals under 650-nm and 578-nm laser irradiation, allowing for the dual-modal assessment of retinal pigment epithelial damage. The same research group also applied a similar CGNP cluster system to visualize the CNV, abnormal blood vessel growth beneath the retinal pigment epithelium, using multimodal photoacoustic microscopy (PAM) and optical coherence tomography (OCT) [106,107].

### 4.2. In Situ Aggregation of GNMs

To fully leverage the distinct advantages of both small and large AuNPs, where the smaller AuNPs exhibit prolonged blood circulation and enhanced cellular or bacterial uptake, while larger AuNPs demonstrate strong NIR absorption, extended retention time, and superior suitability for PA imaging and PTT in deep tissues. A novel strategy has been developed to induce in situ aggregation of AuNPs through internal or external stimuli [100,115,116,117,118,119,120,121,122,123,124,125,126,127,128,129,130,131,132,133,134,135]. Notably, the internal stimuli encompass biological components and microenvironmental factors such as immune cells [116,117], glutathione (GSH) [118,119,120,121], reactive oxygen species (ROS) [122,127,128], pH fluctuations [124,125,126], and various types of ions, molecules, proteins, and/or enzymes (e.g., caspase-3, etc.) [115,129,130]. External stimuli primarily include the chemiluminescence [131], pharmacological agents (such as vascular disrupting agents) [133], ultraviolet light [100,134], and bioorthogonal click-to-release (BCR) reactions [135].

With regard to internal stimuli, Emelianov et al. developed a kind of PAI contrast agent composed of glycol-chitosan-coated AuNPs (short for GC-AuNPs), enabling the noninvasive detection of sentinel lymph node (SLN) metastases [117]. In brief, following peritumoral injection, the GC-AuNPs were initially internalized by immune cells, where they were triggered to aggregate intracellularly [117]. Subsequently, the GC-AuNP-labeled immune cells were transported via lymphatic vessels to SLN. The presence of metastatic lesions influenced the distribution of these labeled immune cells, as metastasis was associated with reduced immune cell accumulation in the SLN. Consequently, the decrease in PA signals intensity was leveraged to monitor and detect SLN metastases using ultrasound-guided photoacoustic (USPA) imaging [117]. More recently, Fan and co-workers designed a GSH-mediated in situ aggregation strategy using PDA-coated AuNPs (Au@PDA) covalently functionalized with mPEG-CONH-ss-NH_2_ (denoted as Au@PDA-ss-PEGm) for combined PAI analysis and tumor therapy [120]. Upon uptake by tumor cells, the high intracellular GSH concentration cleaved the disulfide bonds on the nanoprobe surface, inducing the aggregation of Au@PDA-ss-PEGm. In contrast, the probes remained stable in systemic circulation due to the low or absent GSH levels, making them suitable for intravenous administration. Specifically, the aggregated NPs exhibited enhanced plasmonic coupling, resulting in significantly amplified photothermal (PCEPT) effects and PA signals, thus enabling simultaneous PAI analysis, photothermal therapy, and safe postoperative treatment [120]. More recently, Li et al. developed a gold-based PA nanocontrast agent composed of Au@MnO_2_ NPs (AMOPs), which enabled controllable PAI analysis of tumors triggered by intracellular GSH [121]. The PA signals of AMOPs increased significantly upon interaction with GSH and correlated positively with the AMOPs’ concentration. Due to strong in vitro GSH-responsive PA performance, AMOPs were administered intravenously to mice with 4T1 tumors for in vivo PA imaging. PA signals at the tumor site rose progressively over the first 8 h, peaked at 8 h, then declined gradually by 24 h due to circulation and metabolism. The peak signal at 8 h indicates effective AMOP accumulation in tumors, likely due to the EPR effects [121]. Furthermore, Wang’s group reported a type of approach for inducing the in situ AuNP aggregation via pH-triggered self-assembly to improve tumor targeting and enhance PA imaging and therapeutic precision [123]. To achieve intratumoral acidic pH-responsive assembly, small AuNPs (Au-MUA-TMA) were synthesized by modifying the surface with 11-mercaptoundecanoic acid (MUA) and (10-mercaptodecyl) trimethylammonium bromide (TMA). This dual ligand modification conferred a mixed-charge zwitterionic surface, prolonging blood circulation time. Upon reaching the tumor microenvironment, the acidic pH induced nanoparticle aggregation, thereby extending intratumoral retention, enhancing PA signal output, and improving PAI image quality. Moreover, Song and coworkers engineered caspase-3 enzyme-activatable nanoprobes (namely AuNNP@DEVD-INT20) by specifically modifying nanogapped AuNPs with an AIE molecule (INT20) and DEVD peptides [127]. Upon encountering both GSH and caspase-3 enzyme in target cells, the nanoprobes released INT20 molecules, triggering aggregation-induced emission (AIE). Therefore, in situ self-assembly of AuNPs occurred via crosslinking between sulfhydryl groups and maleimide moieties, suitable for ratiometric PAI analysis of tumors. Remarkably, the nanoprobes also generated substantial ROS under X-ray irradiation, further demonstrating the potential for cancer radiotherapy [122,127,128].

**Figure 6:**
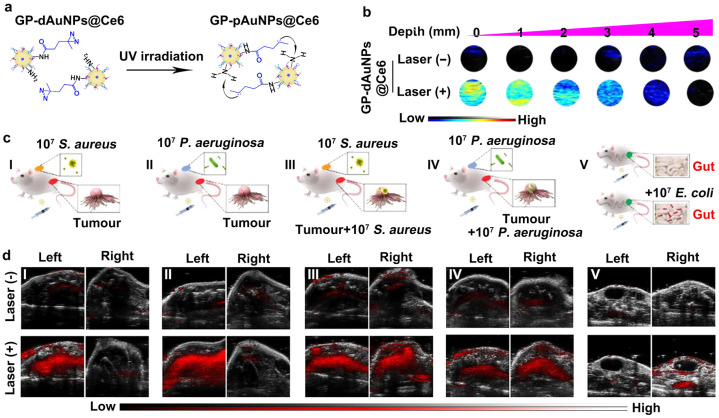
In vivo PAI analysis of bacterial infections in skin, tumor, and gastrointestinal tract using GP-dAuNPs@Ce6 nanoprobes: (**a**) schematic illustration of the mechanism underlying UV irradiation-induced controllable aggregation of GP-dAuNPs@Ce6; (**b**) PA signal intensity of the developed GP-dAuNPs@Ce6 as a function of chicken breast tissue thickness, with and without UV laser irradiation; (**c**) schematic illustration of the experimental models for establishing bacterial infections in the skin, tumor, and gut; and (**d**) in vivo PA images of bacterial infections in skin, tumor, and gastrointestinal tract using GP-dAuNPs@Ce6 nanoprobes. Group I: Tumor (right side) and *S. aureus* (left side); Group II: Tumor (right side) and *P. aeruginosa* (left side); Group III: Tumor + *S. aureus* (right side) and *S. aureus* (left side); Group IV: Tumor + *P. aeruginosa* (right side) and *P. aeruginosa* (left side); Group V: Gut (left side) and Gut + *E. coli* (right side). Reprinted with permission from Ref. [134]. Copyright 2022 Springer Nature.

Further, recent studies on in situ aggregation of AuNPs have demonstrated that ultraviolet light [100,134], pharmacological agents, such as vascular disrupting agents [133], as well as BCR reaction [135], have emerged as simple and effective strategies to induce AuNP aggregation. In 2016, Gao et al. functionalized the surface of AuNPs with PEG5000 ligands terminated with diazirine groups, which are responsive to 405-nm ultraviolet light, to develop the photolabile AuNPs-based nanoprobes [100], designated as PEG-AuNPs. Upon irradiation with a 405-nm laser, covalent crosslinking occurred between diazirine groups on adjacent NPs, resulting in the formation of larger AuNP aggregates. The results demonstrated that ultraviolet light-induced AuNP aggregates exhibited significantly enhanced PA signals and photothermal effects, whereas the original PEG-AuNPs without UV irradiation generated negligible PA signals and minimal photothermal activity [100]. To extend this UV-assisted aggregation strategy for disease diagnosis and treatment, He and colleagues further designed an AuNPs-based nanoprobe, GP-dAuNPs@Ce6, mainly composed of glucose polymer (GP)- and diazirine-conjugated AuNPs loaded with Ce6 [134]. Upon the irradiation of UV light, the developed GP-dAuNPs@Ce6 could significantly gather into the large-sized nanoaggregates, namely GP-pAuNPs@Ce6 (Figure 6a). Due to the UV-induced aggregation of GP-dAuNPs@Ce6, their imaging depth of PA signals increased to above 4 mm, potential for in vivo PAI analysis (Figure 6b). Importantly, these GP-dAuNPs@Ce6 probes were capable of being internalized by various bacteria, including both Gram-negative and Gram-positive species, followed by in situ aggregation upon 405-nm laser irradiation. To further validate the potential for multimodal imaging applications, several kinds of disease models, including bacterial skin infections, intratumoral bacterial colonization, peritonitis, and others, were thus established (Figure 6c). Figure 6d shows the strong fluorescence signals from Ce6 and enhanced PA signals from AuNPs specifically in bacteria-rich regions, while negligible signals were detected in non-infected areas. Collectively, these findings demonstrate that the UV-triggered in situ aggregation approach enhances the feasibility of the small-sized AuNPs for high-performance PA imaging and PTT of diseases such as bacteria-induced diseases [134]. Most recently, Zhang et al. synthesized iminosydnone-lonidamine (ImLND) as a prodrug and selected dibenzocyclooctyne (DBCO) as the trigger for BCR reaction [135]. Based on this design, they further developed a PEGylated AuNPs-based two-component nanoplatform, comprising prodrug-loaded AuNPs-ImLND and tumor-targeting peptide RGD-conjugated AuNPs-DBCO-RGD. Upon reaching the tumor site, AuNPs-ImLND undergoes BCR with DBCO-functionalized AuNPs, leading to the formation of highly photothermally active AuNP nanoaggregates [135]. The concurrent release of the lonidamine enhances therapeutic efficacy by sensitizing cancer cells to PTT [135]. Taken together, the in situ aggregation of GNM integrates the advantages of both small-sized and large-sized nanoparticles, making it highly suitable for in vivo PAI analysis and disease diagnosis.

Overall, pre-formed gold nanoaggregates, such as nanoclusters and assemblies, offer controlled, reproducible properties [103,104,105,106,107,108,109,110,111,112,113,114]. Their size, shape, and structure can be precisely tuned during synthesis, allowing for the optimization of optical properties (e.g., strong NIR absorption) and pharmacokinetics (e.g., circulation time, biodistribution), leading to consistent PA performance. The surface coatings enhance stability, reducing in vivo disassembly and ensuring a stable PA signal over time, essential for reliable imaging and quantification. Their well-defined surfaces also allow for conjugation with targeting ligands, like antibodies or peptides, enabling active targeting and increased PA signal specificity at disease sites. In contrast, the in situ gold nanoaggregation provides high specificity and signal-to-noise ratio through a “switch-on” PA signal that activates only at the target site [115,116,117,118,119,120,121,122,123,124,125,126,127,128,129,130,131,132,133,134,135]. The small AuNPs used in this approach circulate for longer and penetrate more deeply into tumors than larger pre-formed clusters. This method is inherently responsive, detecting biomarkers, such as enzymes (e.g., matrix metalloproteinases) or acidic pH, enabling activatable PA imaging. Therefore, the choice between strategies depends on the application. The pre-formed gold nanoaggregates are robust “always-on” probes ideal for strong, stable contrast. In situ nanoaggregates offer smarter, stimuli-responsive detection with higher target-to-background ratios for sensitive biomarker identification, though less predictably. Future approaches may combine the design control of pre-formed nanostructures with the responsiveness of in situ assembly.

## 5. Challenges and Future Outlook

In summary, various types of the functionalized gold nanoprobes, made of different GNMs, such as AuNPs, AuNRs, AuNFs, AuNSs, AuNTs, AuNShs, have shown significant promise as contrast agents for the PAI detection of diseases. Nevertheless, several challenges remain in achieving in-depth in vivo PAI analysis and effective disease diagnosis in the future. (I) These gold nanoprobes exhibit high signal-to-background ratios, along with excellent imaging resolution and contrast. Their tunable optical properties, strong NIR absorption, and favorable biocompatibility enable high-quality PAI for the detection and diagnosis of cancer, vascular plaques, inflammatory conditions, ocular disorders, and bacterial infections. However, as briefly summarized in Table 1, Table 2 and Table 3, The PAI window of most functionalized gold nanoprobes is located within the NIR-I region (ca. 650–950 nm), with some even confined to the visible light region (ca. 400–700 nm), resulting in limited tissue penetration depth (less than several millimeters). Only a few functionalized gold nanoprobes exhibit absorption in the NIR-II region (ca. 1000–1700 nm), which can demonstrate superior tissue penetration, reaching several centimeters [53]. As a result, compared to current ultrasound imaging analysis, PAI currently offers a more limited tissue penetration depth, due to the tissue penetration capacity of the excitation pulsed laser, which is typically less than 5 cm. This inherent limitation significantly hinders its applicability in meeting the demanding requirements of comprehensive analysis and accurate diagnosis in complex diseases in deep tissues. (II) Drawing on current research into gold nanoprobes, scholarly efforts have primarily focused on enhancing imaging performance. More importantly, the biomedical applications of GNMs in targeted disease therapy remain a key priority, with imaging serving as a complementary tool to validate therapeutic efficacy. Meanwhile, most gold nanoprobes have been employed for PAI analysis ranging from in vitro studies to in vivo experiments in mice and rabbits (Table 1, Table 2 and Table 3). These studies consistently report that gold nanoprobes can exhibit favorable biosafety profiles, demonstrating cell viability above 90% after exposure, with no significant alterations observed in major tissues, organs, or blood parameters. Multiple studies show that functionalized gold nanoprobes selectively target and accumulate at disease sites, with primary clearance through the liver, kidneys, and spleen. As further reported, the cytotoxicity and biocompatibility of GNMs depend on their physicochemical properties and administration routes [136,137,138]. For instance, oral and intraperitoneal delivery are more toxic than intravenous injection, and biocompatibility varies across organs. Cytotoxicity is closely tied to particle size, shape, surface charge, and aggregation state. Despite progress, GNM toxicity findings remain inconsistent in both in vitro and in vivo models. Over the past decades, many nanotherapeutics have gained regulatory approval. However, currently approved FDA formulations mainly use polymeric or liposomal carriers. Recent research has increasingly focused on inorganic and metallic nanoparticles, with GNMs emerging as top candidates. So far, only a few GNM-based formulations are in clinical evaluation [136,137,138]. Nevertheless, the long-term biodistribution, metabolic fate, and immunological implications of functionalized gold nanoprobes have not yet been systematically assessed or fully elucidated. Therefore, to facilitate the clinical translation of these agents, a comprehensive and rigorous safety evaluation, encompassing long-term biodistribution, metabolic pathways, and immune responses, is essential. (III) Gold nanoprobes are susceptible to aggregation and morphological changes in physiological environments, resulting in altered optical properties and reduced PA signal intensity. Further, their long-term structural stability is uncertain, potentially leading to unintended biological interactions. Various surface functionalization strategies have been employed to enhance the stability of gold nanoprobes. For instance, PEG-coated AuNRs demonstrate excellent serum stability, maintaining a consistent hydrodynamic size of ~200 nm over a 24 h incubation period in a fetal bovine serum (FBS)-containing medium. Nevertheless, a systematic and comprehensive evaluation of stability remains essential. (IV) Ligand-mediated active targeting strategies of gold nanoprobes are frequently confronted with in vivo challenges, such as limited tissue penetration, non-specific binding, and protein corona formation, which can shield targeting ligands and compromise targeting specificity.

Consequently, significant research efforts are urgently needed to overcome the current limitations in PAI analysis and diagnosis of diseases. (1) The imaging depth can be significantly enhanced by optimizing the pulsed laser irradiation configuration, such as employing ring-shaped or multi-angle illumination, in combination with the strategic use of optimal wavelengths in the NIR-II region, particularly in the NIR-IIb subwindow (~1500–1700 nm), which offers superior tissue penetration, and the integration of low-frequency ultrasonic detectors. (2) Deep learning (DL) and artificial intelligence (AI) improve deep-tissue PAI quality by enhancing image reconstruction, noise suppression, resolution, artifact reduction, and multimodal fusion. Using data-driven models, they capture complex imaging mechanisms and overcome limitations of traditional methods. DL enables accurate reconstruction from undersampled data by learning the mapping between raw signals and high-quality images, reducing sampling constraints. AI suppresses noise and artifacts, lowering background interference. With reinforcement learning, the system adjusts key parameters, such as excitation wavelength, laser pulse energy, and detection angle, in real time to maximize signal strength and minimize noise. (3) Smart gold nanoprobes that activate PA signals in response to different disease biomarkers, such as altered pH or overexpressed enzymes and proteins, improve signal-to-noise ratio and diagnostic specificity, enabling more accurate early detection. (4) To minimize potential long-term in vivo toxicity, it is essential to develop smart and biodegradable gold nanoclusters that degrade into excretable byproducts after their use in bioimaging applications, particularly yielding ultrasmall nanoparticles (<6 nm) capable of renal clearance. (5) Next-generation surface coatings enhance colloidal stability, reduce protein corona formation, and improve stealth properties, leading to better in vivo performance and longer circulation of nanomedicines. In conclusion, by transitioning from conventional contrast agents to intelligent, biodegradable, and multifunctional theranostic systems, we can fully realize their potential for precise disease diagnosis and therapeutic interventions.

## Figures and Tables

**Table 1:** Comparison of photoacoustic performance, multifunctionality, and disease imaging analysis of different Janus gold nanoprobes.

Nanoprobes	Types of GNMs	One Side	Another Side	Models	Imaging Window	Ref.
J-Au-CS	AuNRs	Chitosan	Rigid AuNRs	Tumors/Mice	1064 nm	[45]
MnO_2_-AuNR-Ppa	AuNRs	MnO_2_	Ppa	Tumors/Mice	1250 nm	[46]
AuNR@DCNP@PEG	AuNRs	SiO_2_/DCNP	Bare AuNRs	Tumors/Mice	1250 nm	[47]
AuPANI NDs	AuNDs	PANI	NOTA	Tumors/Mice	1064 nm	[48]
GSJNPs	AuNPs	SiO_2_	Bare AuNPs	Lymph node/mice	700 nm	[50]
JNCPs	AuNPs	IR Dyes	AuNPs	Tumors/Mice	690/825 nm	[51]

**Table 2:** Comparative analysis of photoacoustic performance, shell configurations, and disease imaging capabilities of various functionalized gold nanocomposite-based nanoprobes.

Nanoprobes	Types of GNMs	Shell	Function	Models	Imaging Window	Ref.
AuNRs/PDA	AuNRs	PDA	Enhancing PA performance	Not mentioned	750–950 nm	[54]
Gold/melanin probes	GNMs	PDA	Enhancing PA performance	Intestine/Mice	764 nm	[55]
AP/p53–HA	AuNRs	HA	Tumor targeting	Tumors/Mice	815 nm	[58]
AuNSPHs	AuNSs	HA	Tumor targeting	Tumors/Mice	850 nm	[60]
LHMSiO_2_−AuNR	AuNPs	SiO_2_	Enhancing PA performance	Not mentioned	750–800 nm	[65]
Au@SiO_2_	AuNRs	SiO_2_	Enhancing PA performance	Not mentioned	500 nm	[67]
Au/AgNRs	AuNRs	Ag	Quenching PA signals	Wound/Mice	800 nm	[71]
PTA NRs	AuNRs	Pd	Enhancing PA performance	Tumors/Mice	800–850 nm	[72]

**Table 3:** Comparative analysis of the photoacoustic performance, aggregation form/response sites, and disease imaging capabilities of different gold nanoaggregate probes.

Nanoprobes	Types of GNMs	Aggregation form	Response Sites	Models	Imaging Window	Ref.
CGNP clusters-RGD	AuNPs	Chain-like	No	CNV/Rabbit	578/650 nm	[106]
PNIPAM-AuNR	AuNPs	Spherical	No	Tumors/Mice	1064 nm	[109]
AuNNPs-Ag_2_S Ve	AuNRs	Spherical	pH (PA OFF)	Tumors/Mice	900–1000 nm	[112]
GC-AuNPs PEG-AuNPs	AuNPs	Nanoclusters	Cells	Metastatic lymph node/mice	680–970 nm	[117]
AMOPs	AuNPs	Nanoclusters	GSH	Tumors/Mice	NIR-I	[121]
Au–MB–PEG	AuNPs	Nanoclusters	ROS	Tumors/Mice	700–900 nm	[122]
Au-MUA-TMA	AuNPs	Nanoclusters	pH	Tumors/Mice	NIR-I	[125]
AuDPFC	AuNPs	Nanoclusters	Spermine	Tumors/Mice	670 nm	[129]
GP-dAuNPs@Ce6	AuNPs	Nanoclusters	UV light	Bacteria/Mice	700–900 nm	[134]
AuNPs-ImLND AuNPs-DBCO-RGD	AuNPs	Nanoclusters	BCR reaction	Tumors/Mice	NIR-I	[135]

## Data Availability

No new data were created or analyzed in this study.
